# Rennet-Induced Gelation Properties of Freeze-Dried Micellar Casein Powder: Influence of Pre-Freezing Temperature

**DOI:** 10.3390/gels12030265

**Published:** 2026-03-22

**Authors:** Chuang Dong, Yun Chen, Lin Yang, Weibo Zhang, Shengbo Yu, Pengjie Wang, Zhishen Mu, Chong Chen

**Affiliations:** 1 Department of Nutrition and Health, China Agricultural University, Beijing 100193, China; 15076090410@163.com (C.D.); wpj1019@cau.edu.cn (P.W.); 2 National Enterprise Technology Center, Inner Mongolia Mengniu Dairy (Group) Co., Ltd., Hohhot 011500, China; chenyun@mengniu.cn (Y.C.); yushengbo@mengniu.cn (S.Y.); 3 Global R&D Innovation Center, Inner Mongolia Mengniu Dairy (Group) Co., Ltd., Hohhot 011500, China; 4 Faculty of Food Science, Xizang Agricultural and Animal Husbandry University, Linzhi 860000, China; yanglin@xza.edu.cn; 5 Department of Food Science and Technology, Faculty of Science, National University of Singapore, Singapore 117542, Singapore; wbzhang@nus.edu.sg

**Keywords:** pre-freezing temperature, casein micelles, rennet-induced gel

## Abstract

Drying significantly influences the quality of micellar casein (MC) powder. This study investigated the effects of three pre-freezing temperatures (−20 °C, −80 °C, and liquid nitrogen) prior to freeze drying on the structure and rennet-induced gelation properties of MC powder. The results showed that as the pretreatment temperature decreased, the degree of disruption to the secondary and tertiary protein structures was reduced, while the particle size gradually increased. In terms of rennet-induced gel properties, the untreated raw MC consistently outperformed all MC powder samples. Among the MC powders, the sample pre-frozen at −80 °C and then freeze-dried (FD-80) exhibited the highest gel strength and a relatively shorter rennet coagulation time. The observed microstructures of the rennet-induced gel were consistent with the rheological results, showing that samples with smaller particle sizes formed more regular and compact gel networks. In conclusion, the MC powder prepared via pre-freezing at −80 °C and then freeze-drying better preserved protein structure and demonstrated superior rennet-induced gel properties, which were closely related to particle size. This study provides theoretical insights for the application of MC powder in products such as cheese, processed cheese, and protein-fortified foods.

## 1. Introduction

Micellar casein (MC), consisting primarily of casein micelles and water, is obtained from skim milk by separating casein micelles from whey proteins, lactose, and minerals via microfiltration [1,2]. MC has garnered significant attention in the food processing and nutrition fields owing to its favorable functional properties (e.g., emulsifying and gelling capabilities) and nutritional attributes (such as amino acid supplements) [3,4,5]. MC is available in various forms, including concentrates and dried powders. Concentrates are bulky and have poor microbial stability, making them not conducive to long-term storage. Due to cost efficiency, transportation, and storage, MC is often utilized in powder form. However, the structure and functionality of MC powder can be altered by temperature variations and dehydration during the drying process, thereby affecting powder quality, such as solubility and gelation properties [6].

Freeze drying and spray drying are the main methods for producing MC powder [7]. It has been shown that freeze-drying (FD) causes minimal damage to milk proteins, preserving their functional properties and bioactive components effectively [8]. The physicochemical properties, microstructure, and proteomic profiles of FD and spray-dried (SD) milk powders from bovine, caprine, and equine sources were investigated. The results indicated that FD powders exhibited higher internal porosity, better rehydration properties, and elevated levels of immunity-related proteins compared to SD powders [9]. It has also been shown that the production of camel milk powder using low-temperature vacuum drying exhibited significantly higher moisture content and solubility than that using spray drying [10]. Another study employed SD and FD techniques to process goat milk powder and showed that the vibration patterns of various characteristic peaks in the mid-infrared spectra were essentially identical, but differences in absorption intensity were observed in powders. The signal intensity of specific components in SD milk powder was higher than that in FD milk powder, with observed differences in the microstructure between these two powders [11]. Furthermore, the pre-freezing method significantly influences protein structure and product quality during freeze-drying. One study evaluated the effects of conventional freezing (−20 °C), ultra-low temperature freezing (−80 °C), and freeze-drying on human milk protein structure. It was found that freezing at −20 °C altered protein secondary structure, reduced free amino nitrogen content, and decreased in vitro digestibility [12]. The aforementioned study investigated the effects of pre-freezing methods and drying methods on the physicochemical properties and functional characteristics of milk proteins. However, research on how pre-freezing methods influence the structure of milk proteins and the patterns of their impact on functional properties remains relatively limited.

Rennet-induced coagulation is one of the most important functional properties of casein, which serves as the fundamental structural component of cheese [4,13]. During cheese making, rennet acts on κ-casein located on the surface of casein micelles, cleaving it at the Phe^105^-Met^106^ bond and releasing casein glycomacropeptide into the whey [14]. Then, the casein micelles begin to aggregate via hydrophobic interactions and calcium bridging, forming a three-dimensional gel network. In this process, casein constitutes the primary gel matrix. Given its high casein content, MC powder has promising applications in cheese production to fortify milk or as an alternative for milk for cheese making [15], enhancing cheese yield and promoting the development of desirable flavor and texture [15,16]. Currently, there is relatively limited research on the gelation properties of MC powder. Less structural damage to proteins by FD treatment and the importance of pre-freezing conditions on protein structure and functional properties have also been emphasized. However, the impact of pre-freezing temperature prior to FD on the structure and rennet-induced gelation properties of MC powder remains unclear.

This research aimed to investigate the influence of different pre-freezing methods on the rennet-induced coagulation characteristics of MC powder. In this study, MC concentrate was subjected to three pre-freezing treatments: −20 °C, −80 °C, and liquid nitrogen, followed by FD to obtain different MC powders. Then, the protein structure of MC powders, rheological properties and microstructure of rennet-induced casein gels were analyzed by dynamic light scattering, fluorescence spectroscopy, Fourier transform infrared spectroscopy (FTIR), rheometer, and scanning electron microscope (SEM). This research provides theoretical insights to support the application of MC powder in cheese manufacturing.

## 2. Results and Discussion

### 2.1. Composition of MC Powder

The compositions of MC powders obtained using different pre-freezing methods are presented in Table 1. All three dried samples exhibited protein contents above 80% and moisture contents below 5% (*w*/*w*), meeting the standard for MC powders (protein content should be ≥80% *w*/*w*) [15]. As shown in Table 1, no significant differences were observed between MC powder samples in moisture and calcium content (*p* > 0.05), while significant differences were found in protein and phosphorus content (*p* < 0.05). The pH of MC was higher than skim milk, because MC is typically produced by concentrating skim milk 2 to 3-fold via microfiltration, a process during which the pH gradually increases [17]. The resulting elevated pH is considered one of the key factors contributing to the favorable heat stability and buffering capacity of MC, as it enhances the negative charge on the surface of casein micelles. This increased electrostatic repulsion prevents the aggregation and precipitation of micelles during heating [18].

### 2.2. FTIR Spectroscopy

**Figure 1:**
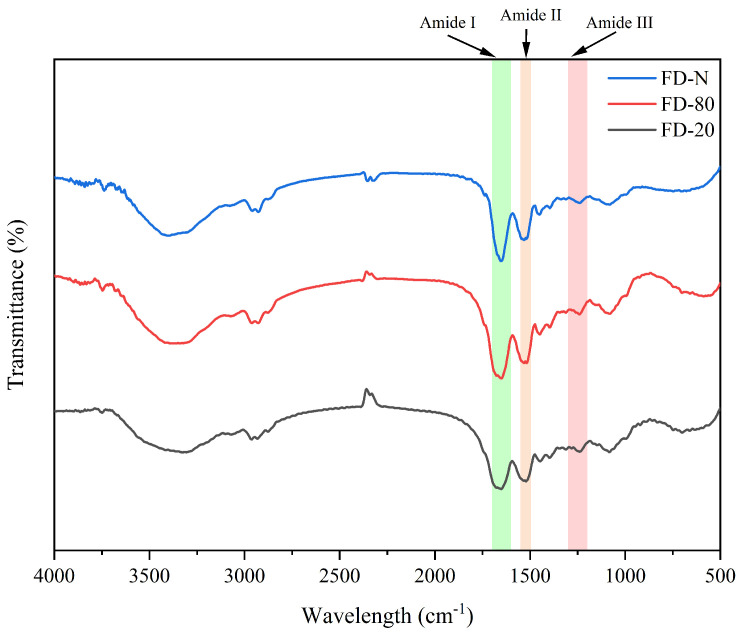
Effect of pre-freezing temperature on the FTIR spectra of MC powder. Among them, FTIR stands for Fourier-transform infrared spectroscopy, MC refers to micellar casein concentrate, while FD-20, FD-80, and FD-N are samples freeze-dried after pre-freezing at −20 °C, freezing at −80 °C, and liquid nitrogen treatment, respectively.

FTIR spectroscopy is an effective technique for analyzing changes in protein secondary structure. The amide I band (1700–1600 cm^−1^) originates primarily from the stretching vibration of C=O bonds, while the amide II band (1600–1500 cm^−1^) is mainly attributed to the combined contributions of C-N stretching and N-H bending vibrations [19]. As shown in Figure 1, no significant changes in peak shape or position were observed within the amide II region, which may be related to the fact that the MC powder consists mainly of casein micelles. Therefore, this study focused on the amide I band as the key region for analyzing changes in protein secondary structure in MC powder. It can be seen from Figure 1 that alterations in peak position and shape in the amide I region indicate noticeable changes in protein secondary structure [20]. Thus, pre-freezing treatment significantly affected the FTIR spectra of the MC powder. The FD-N exhibited a relatively sharp amide I band, which may be attributed to the rapid freezing in liquid nitrogen that preserves protein native conformation and minimizes ice crystal-induced damage [21]. In contrast, the amide I bands of FD-20 and FD-80 were broader and smoother, with the FD-20 displaying the broadest profile. This results from slower freezing at −20 °C, which promotes the formation of larger ice crystals and generates greater mechanical stress, leading to increased structural heterogeneity, higher conformational flexibility, and greater disorder in the protein arrangement [22].

### 2.3. Fluorescence Spectroscopy

**Figure 2:**
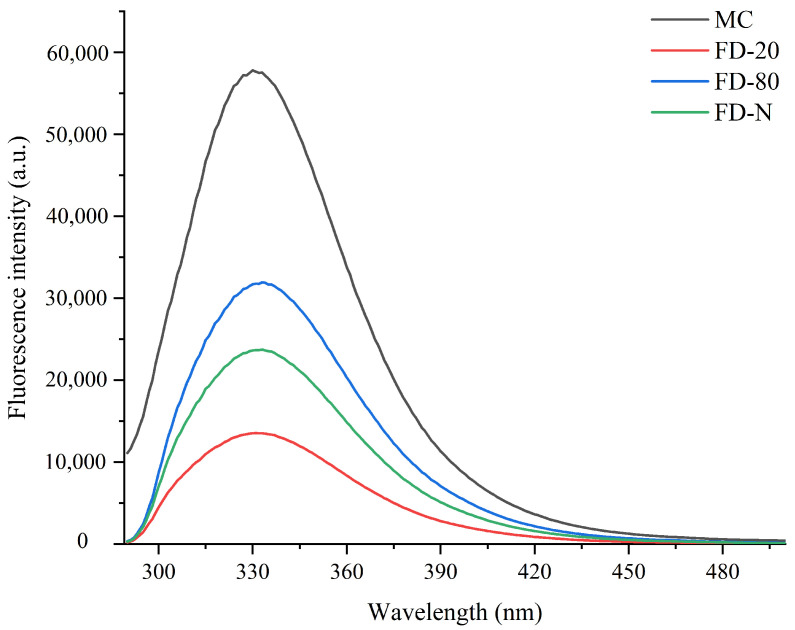
Effect of pre-freezing temperature on the fluorescence spectra of MC powder. Among them, MC refers to micellar casein concentrate, while FD-20, FD-80, and FD-N are samples freeze-dried after pre-freezing at −20 °C, freezing at −80 °C, and liquid nitrogen treatment, respectively.

The maximum fluorescence spectrum of tryptophan can indicate the relative position of tryptophan residues within a protein and is used as an indicator of changes in the higher-order structure of proteins. Therefore, fluorescence spectroscopy is an effective technique for assessing the unfolding extent of a protein’s tertiary structure [2,23]. As shown in Figure 2, among all the tested samples, the original MC sample without drying exhibited the highest fluorescence intensity, indicating that the tertiary structure of its casein micelles was the most intact, with tryptophan residues mainly residing in a relatively hydrophobic environment. In contrast, all freeze-dried MC powder samples showed varying degrees of reduction in fluorescence intensity, suggesting that the drying process had a significant impact on the tertiary structure of the protein. Upon further observation of samples subjected to different pre-freezing treatments, it was found that the FD-80 sample maintained a relatively high fluorescence intensity after drying, which was significantly higher than that of FD-20 and FD-N. The results indicate that rapid freezing to −80 °C better preserves the natural conformation of casein, effectively protecting the internal tryptophan residues during the drying process and resulting in less structural damage. This is possibly due to the formation of fine and uniform ice crystals during rapid freezing, which reduces the mechanical stress-induced disruption to the spatial structure of the protein. In contrast, the FD-20 experienced slow freezing that promoted the formation of large ice crystals. The resulting mechanical stress likely disrupted the protein tertiary structure, leading to reduced fluorescence intensity. These results indicated that both pre-freezing treatment and dry-freezing significantly affected the structure of MC powder.

### 2.4. Particle Size

**Figure 3:**
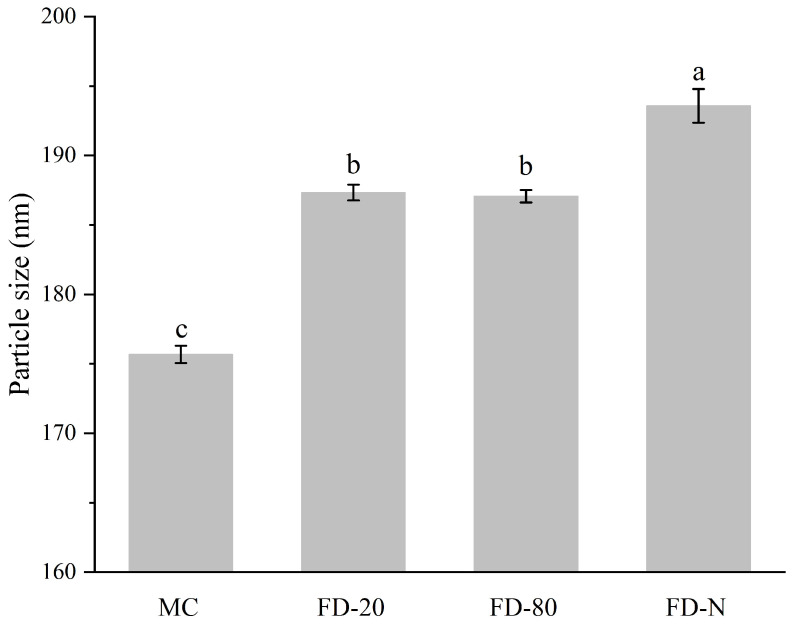
Effect of pre-freezing temperature on particle size of MC powder. Among them, MC refers to micellar casein concentrate, while FD-20, FD-80, and FD-N are samples freeze-dried after pre-freezing at −20 °C, freezing at −80 °C, and liquid nitrogen treatment, respectively. Different lowercase letters indicate significant differences.

As shown in Figure 3, the initial MC solution showed the smallest particle size (175.67 ± 0.63 nm). The particle size of all MC powders after hydration increased compared to the original MC, suggesting the dry process changed the structure of the casein micelle [6]. There was no significant difference in the particle size of the reconstituted solutions between FD-20 and FD-80 powders (*p* > 0.05), while the FD-N powder showed the largest particle size in its reconstituted solution (193.57 ± 1.21 nm). The increase in particle size may affect the long-term physical stability of the solution, making it more prone to sedimentation or stratification during storage. Additionally, the polydispersity index (PDI) values for MC, FD-20, FD-80, and FD-N were 0.115 ± 0.003, 0.135 ± 0.005, 0.123 ± 0.004, and 0.120 ± 0.002, respectively. With PDI values ranging from 0.1 to 0.15, the system exhibits good homogeneity and stability. Thus, the particle sizes of casein micelles from the MC powder increased as the temperature of pre-freezing treatment decreased. Current research indicates that low-temperature conditions may induce partial solubilization of colloidal calcium phosphate within the micelles, leading to the weakening of internal binding forces within casein micelles [24], leading to swelling of the casein micelle and an increase in particle size [25]. The particle size also partly explains why the fluorescence intensity of FD-N is lower than that of FD-80. The swelling of casein micelles exposes more hydrophobic groups, resulting in fluorescence quenching.

### 2.5. Rheological Properties

#### 2.5.1. Time Sweep

**Figure 4:**
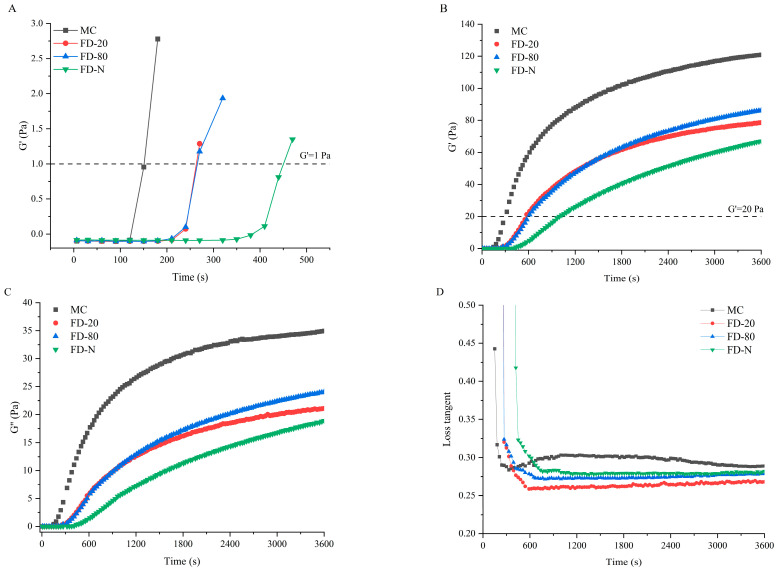
Time-sweep of rennet-induced gelation properties of MC powder. Among them, MC refers to micellar casein concentrate, while FD-20, FD-80, and FD-N are samples freeze-dried after pre-freezing at −20 °C, freezing at −80 °C, and liquid nitrogen treatment, respectively. (**A**) Enlarged View of G′ under Time Sweep; (**B**) G′ under Time Sweep; (**C**) G″ under Time Sweep; (**D**) Tanδ under Time Sweep.

The influence of different pre-freezing temperatures on the rennet-induced gelation properties of MC powder was investigated using rheology. Figure 4 presents the time-sweep curves of rennet-induced gels for different MC powders. The rennet coagulation time (RCT), defined as the time when the storage modulus (G′) exceeds 1 Pa, is a key parameter for evaluating the gelation characteristics of casein micelles [19,26]. As shown in Figure 4A, all MC powders showed prolonged RCT compared to the original MC. No significant difference (*p* > 0.05) was observed between the RCT of FD-20 and FD-80, while the FD-N displayed the longest RCT. The cutting time is another critical parameter that influences final cheese quality, which is defined as the time when G′ reaches 20 Pa [27]. As shown in Figure 4B, the trend in cutting time aligns with that of RCT. These results suggested that pre-freezing temperature significantly affected the rennet-induced gelation properties of MC powder. The prolongation of RCT in all dried samples may be due to the masking of the cleavage site of κ-CN during the drying process, thereby reducing the enzymatic cleavage efficiency and resulting in an increase in RCT. Among them, the freezing rates of FD-20 and FD-80 were lower than that of FD-N. As a result, FD-N retained more κ-CN on the micelle surface, requiring a longer reaction time for enzymatic cleavage, which further extended its RCT.

Additionally, the drying process disrupted the micellar structure, and subsequent reconstitution failed to fully restore the native structure [28], leading to lower G′ and loss modulus (G″) in the gels of the MC powders than that of the original MC. The G′ and G″ of the gels of MC powders were consistent with the results of time-sweep and casein micelle size. Although no significant difference (*p* > 0.05) was found in particle size between FD-20 and FD-80, the gels of FD-80 showed higher G′ and G″ than FD-20. This may be attributed to the rapid freezing at −80 °C, which better preserved the original micellar structure (Figure 1 and Figure 2). In contrast, the slower freezing at −20 °C promoted the growth of large ice crystals, resulting in mechanical damage to the structure of casein micelle [25], thereby reducing G′ and G″. The FD-80 showed the best gelation properties among the three MC powders. The difference in gelation properties can be explained by the changes in particle size of casein micelles. In this study, smaller particle sizes of casein micelle corresponded to lower RCT and higher G′ and G″ values. It has been reported that casein micelle size can influence its gelation properties; the smaller casein micelles lead to shorter coagulation times and higher storage modulus [13,29]. Furthermore, Zhang et al. suggested that a reduction in micellar calcium may lead to longer RCT and a looser gel network [30], explaining the longest RCT and cutting time, and the lowest storage modulus of FD-N due to partial dissociation of micellar calcium [24].

Loss tangent Tanδ (G″/G′) is a parameter in rheology used to characterize the relaxation behavior of a material within the testing timescale, reflecting the dynamic nature of interactions within the gel [31]. Changes in its value can directly indicate potential alterations in the type, strength, and number of interactions within the gel network. As illustrated in Figure 4D, the initial MC exhibited a higher Tanδ than the MC powder, suggesting that its gel may be more prone to syneresis, displaying stronger viscous characteristics and relatively weaker elastic behavior. This could be attributed to possible partial casein aggregation during the microfiltration process of the initial MC, which increases system viscosity [32]. In contrast, the MC powder undergoes drying treatment that disrupts the cross-linked structure between caseins, leading to a lower Tanδ in the resulting gel.

#### 2.5.2. Frequency Sweep

**Figure 5:**
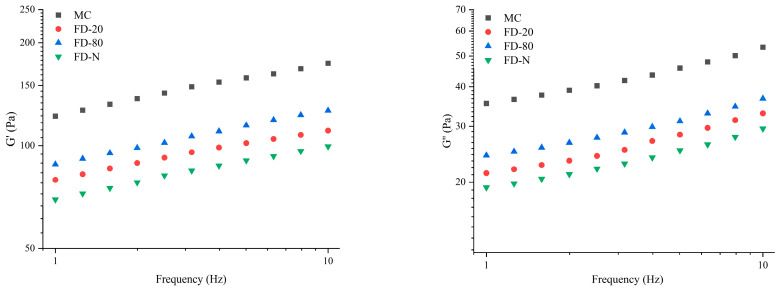
Frequency-sweep of rennet-induced gelation properties of MC powder. Among them, MC refers to micellar casein concentrate, while FD-20, FD-80, and FD-N are samples freeze-dried after pre-freezing at −20 °C, freezing at −80 °C, and liquid nitrogen treatment, respectively.

As shown in Figure 5, a frequency sweep was performed immediately after the time sweep test. As the frequency increased, both G′ and G″ of all samples gradually rose. Among all samples, MC displayed the highest G′ and G″ values. After drying, the G′ and G″ of all samples exhibited varying degrees of reduction, following the order of FD-80, FD-20, and FD-N, indicating that the drying process disrupted the gel properties of the samples. Furthermore, the rennet-induced gels of all samples exhibited consistent trends in G′ and G″ changes, with G′ consistently higher than G″, indicating that elastic behavior dominated in the gels. By fitting the linear relationship between G′ and frequency on a logarithmic scale, the results showed that the slopes of the four samples ranged from 0.14 to 0.16 (R^2^ = 0.99). This indicates that the gels have weak frequency dependence, which may be related to bond rupture and reformation as well as intermolecular interactions within the protein gels [33].

### 2.6. Scanning Electron Microscopy

**Figure 6:**
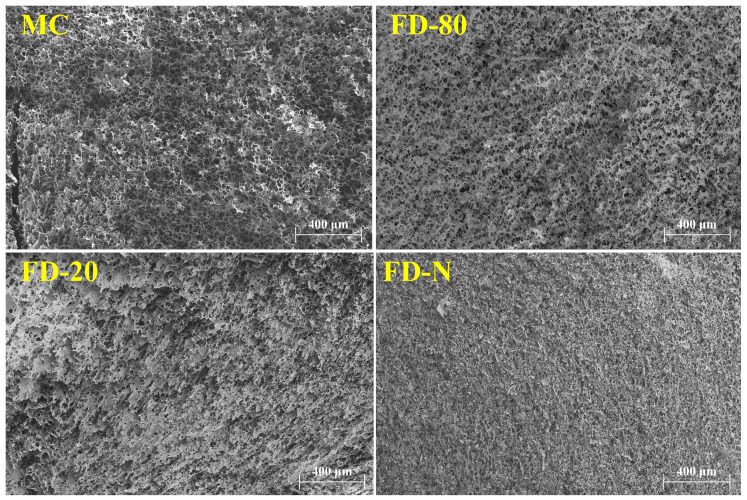
SEM images of rennet-induced gels of MC powder. Scale bars are 400 μm. Among them, SEM stands for scanning electron microscopy, MC refers to micellar casein concentrate, while FD-20, FD-80, and FD-N are samples freeze-dried after pre-freezing at −20 °C, freezing at −80 °C, and liquid nitrogen treatment, respectively.

The microstructure of rennet-induced casein gels was observed using SEM. As shown in Figure 6, the MC sample displayed a highly porous, dense, and uniform gel network, which typically facilitates water retention as well as enhances gel strength. In contrast, the gels from the MC powders exhibited sheet-like cross-linked structures and an overall irregular architecture. This reflects its limited ability to reorganize during the gelation process, which may affect the functional properties of the gel. However, the rennet-induced gels of FD-80 retained a relatively higher porosity and more uniform structure among the three MC powders, which may contribute to its higher G′. Although the gels of FD-N appeared dense, their internal arrangement was disordered and irregular, resulting in a mechanically fragile texture. Our results were consistent with previous studies, which have reported that smaller casein micelles tend to form denser and stronger gel networks [13,34,35].

## 3. Conclusions

This study investigated the effects of three pre-freezing temperatures on the structure and rennet-induced gelation properties of MC powder. The results indicated that the basic composition of MC powder was not significantly altered by the pre-freezing temperature. However, the micellar structure, protein conformation, and gelation properties were markedly affected. Among the treatments, the MC powder pre-freezing at −80 °C best preserved the protein structure and maintained a smaller particle size. Then, the rennet-induced gels of FD-80 showed shorter RCT and higher storage modulus, along with more uniform and denser microstructure. Furthermore, the pre-freezing temperature affected the rennet-gelation characteristics of MC powder by modulating casein micelle size. Specifically, the variation in casein micelle size significantly influences the rennet-induced gelation properties, with smaller micelle sizes favoring a shorter rennet coagulation time (RCT) and promoting the formation of a denser three-dimensional network. Meanwhile, results from FTIR and fluorescence spectroscopy analyses further confirm that pre-freezing at −80 °C minimizes mechanical damage to the protein’s secondary and tertiary structures caused by ice crystals, thereby preserving more of its natural conformation, this provides a structural foundation for the subsequent formation of the gel network. This study provides a theoretical basis for optimizing the coagulation process and gel properties of cheese products, while also proposing practical and feasible solutions for the quality control and targeted processing of functional milk protein ingredients.

## 4. Materials and Methods

### 4.1. Materials

Rennet (average activity 200 IMCU/mL) was purchased from CHR. Hansen (Beijing) Trading Co., Ltd. (Beijing, China). The MC concentrate was kindly provided by Mengniu Dairy (Group) Co., Ltd. (Hohhot, China). Potassium bromide was purchased from Shanghai Macklin Biochemical Co., Ltd. (Shanghai, China). All the other reagents used were of analytical purity.

### 4.2. Sample Preparation

The MC concentrate was subjected to three different pre-freezing treatments: at −20 °C for 12 h, at −80 °C for 12 h, or in liquid nitrogen for 10 min. All samples were subsequently freeze-dried (LGJ-10 freeze-dryer, Beijing Sihuan Sailing Technology Co., Ltd., Beijing, China) for 48 h to obtain MC powders. These powders were designated as FD-20, FD-80, and FD-N, respectively, based on the pre-freezing treatments at −20 °C, −80 °C, and liquid nitrogen.

### 4.3. Physicochemical Indexes of MC Powder

The protein content of MC powder was determined using the Kjeldahl method. Moisture content was analyzed using a rapid halogen moisture meter (VM-E10, Jiangsu Vicometer Instrument Co., Ltd., Taizhou, China). Calcium (Ca) and phosphorus (P) contents were determined by inductively coupled plasma optical emission spectrometry (ICP-OES, Thermo ICAP 7200, Thermo Fisher Scientific, Waltham, MA, USA). The pH values of the MC concentrate and the reconstituted solution of MC powder were measured using a pH meter (PHS-3C, Shanghai INESA Scientific Instrument Co., Ltd. Shanghai, China).

### 4.4. Fourier Transform Infrared Spectroscopy of MC Powder

An FTIR spectrometer (INVENIO S spectrometer, Bruker Technology Co., Ltd., Bremen, Germany) was used to analyze the structure of MC powder. The samples were mixed with potassium bromide (KBr) at a ratio of 1:100 (*w*/*w*), ground thoroughly, and pressed into thin pellets for measurement. Spectra were recorded in the range of 4000–500 cm^−1^ at a resolution of 4 cm^−1^, using a pure KBr pellet as the background.

### 4.5. Fluorescence Spectroscopy of MC Powder

Fluorescence spectra of MC powder were recorded using a fluorescence spectrophotometer (RF-5301PC, Shimadzu Corporation, Shimadzu, Japan). MC powder was diluted to a final concentration of 1 mg/mL with deionized water and scanned at room temperature. The excitation wavelength was set at 280 nm, and the emission spectrum was collected from 290 to 500 nm.

### 4.6. Particle Size of Casein Micelle from MC Powder

The particle size of casein micelle suspensions was measured at 25 °C using a Zetasizer (ZS3600, Malvern Instruments Ltd., Malvern, UK) [36]. Before the measurement, MC powder was dispersed in deionized water to obtain casein micelle suspensions with a final casein concentration of 2.5% (*w*/*v*). The solution was diluted 100-fold with deionized water prior to analysis. For each measurement, 1 mL of the diluted sample was used. The refractive indices were set at 1.33 for the dispersant and 1.45 for the sample.

### 4.7. Rheological Properties of Rennet-Induced Gels

Rennet-induced coagulation properties were characterized using a rotational rheometer (AR1500ex, TA Instruments, New Castle, DE, USA) according to the method, with some modifications [37]. Prior to testing, the 2.5% (*w*/*v*) casein micelle suspension was equilibrated at 32 °C for 30 min in a water bath. Then, rennet was diluted 10-fold with deionized water and added to 20 mL of the suspension to achieve a final concentration of 0.04 IMCU/mL. The mixture was stirred for 30 s and loaded onto the rheometer plate. Finally, time sweep test and frequency sweep were performed. The time sweep test was conducted at 32 °C for 1 h with a constant strain amplitude of 1% and a frequency of 1 Hz. Followed by the frequency sweep test, the strain was maintained at 1% while the frequency was varied from 1 to 10 Hz at a constant temperature of 32 °C.

### 4.8. Scanning Electron Microscopy of Rennet-Induced Gels

The microstructure of rennet-induced gels was examined using SEM (Thermo Scientific Apreo 2S, Thermo, Waltham, MA, USA). Gel samples were rapidly frozen in liquid nitrogen and subsequently freeze-dried. The dried samples were fractured and sputter-coated with gold. Micrographs were taken at a magnification of 200× with an accelerating voltage of 5 keV.

### 4.9. Data Analysis

All experiments were performed in triplicate, and results were expressed as mean ± standard deviation. Data were analyzed using SPSS 24 software (Version 24, SPSS Inc., Chicago, IL, USA). One-way analysis of variance (ANOVA) was employed to determine significant differences. *p* < 0.05 was considered statistically significant.

## Figures and Tables

**Table 1:** Composition of MC and MC powder.

	MC	FD-20	FD-80	FD-N
Total protein (g/100 g)	8.60 ± 0.06 ^c^	83.40 ± 0.20 ^a^	82.20 ± 0.20 ^b^	82.05 ± 0.05 ^b^
Moisture content (g/100 g)	90.10 ± 0.10 ^a^	3.67 ± 0.27 ^b^	3.13 ± 0.48 ^b^	3.70 ± 0.10 ^b^
Total calcium (mg/100 g)	219.0 ± 2.0 ^b^	2695.0 ± 75.0 ^a^	2685.0 ± 75.0 ^a^	2695.0 ± 35.0 ^a^
Total phosphorus (mg/100 g)	147.0 ± 2.0 ^c^	1885.0 ± 5.0 ^a^	1855 ± 15.0 ^b^	1835.0 ± 5.0 ^b^
Suspension solution pH	7.17 ± 0.01 ^a^	7.22 ± 0.04 ^a^	7.25 ± 0.02 ^a^	7.20 ± 0.01 ^a^

Different superscripts within the same row represent significant differences (*p* < 0.05). Additionally, MC refers to micellar casein concentrate, while FD-20, FD-80, and FD-N are samples freeze-dried after pre-freezing at −20 °C, freezing at −80 °C, and liquid nitrogen treatment, respectively.

## Data Availability

The original contributions presented in this study are included in the article. Further inquiries can be directed to the corresponding authors.
